# PP26 Experience In Mitigating Budgetary Impacts Of High Cost Innovations In Uruguay’s Healthcare System Through Partial Disinvestment Actions

**DOI:** 10.1017/S0266462325102456

**Published:** 2025-12-29

**Authors:** Daniel Pedrosa, Diego Scasso, Valeria Cabrera, Eliana Lanzani, Krizia Osterkamp, María Porcelli

## Abstract

**Introduction:**

In 2024, 24 therapeutic sites were expanded within the universal coverage provided by the National Resources Fund (FNR) for all users of the National Integrated Health System (SNS), with an annual investment of USD30 million, mainly for the expansion of immunotherapy coverage. The FNR promotes the development of financial coverage regulations, discouraging the use of widely used drugs that could be subject to partial disinvestment within the SNS.

**Methods:**

An increase in the FNR’s benefits basket was proposed in 2024, focusing on technologies with proven evidence and innovation. This initiative linked health technology evaluations, social and judicial demands, and budgetary impact analyses to determine the required investment. The proposed methodology targeted therapeutic areas where older comparators within the benefits basket could have their indications reduced in consultation with clinical experts, leading to a decrease in investment costs. The relationship between the cost of investment and the cost of disinvesting current technologies was calculated, enabling comparisons between technologies for disinvestment.

**Results:**

Within the SNS, the FNR’s high cost benefits basket proposed the modification of 12 treatments across 24 therapeutic sites for 2024. Of these 12 treatments, eight had active ingredients already funded by the FNR. In developing the regulations and conducting the budgetary impact analysis, a partial disinvestment of previously funded active ingredients was considered. The cost variation associated with disinvestment was quantified by comparing the costs of investment to the costs of disinvestment. The budgetary quantification showed variations ranging from 1.37 percent of the investment value in lung cancer to 24.5 percent in hepatocarcinoma, with significant heterogeneity among the different services.

**Conclusions:**

To ensure the sustainability of healthcare systems, it is necessary to have dynamic benefits baskets that facilitate a constant flow of disinvestment alongside investments. The quantification of technologies to be disinvested in the FNR’s experience should not be considered a marginal expense. It is prudent to implement methodologies that encourage disinvestment, thus ensuring the system’s sustainability.

